# 2-{(1*E*)-[(*E*)-2-(2,6-Di­chloro­benzyl­idene)hydrazin-1-yl­idene]meth­yl}phenol: crystal structure, Hirshfeld surface analysis and computational study

**DOI:** 10.1107/S2056989019012349

**Published:** 2019-09-10

**Authors:** Rohit B. Manawar, Mitesh B. Gondaliya, Manish K. Shah, Mukesh M. Jotani, Edward R. T. Tiekink

**Affiliations:** a Chemical Research Laboratory, Department of Chemistry, Saurashtra University, Rajkot - 360005, Gujarat, India; b Department of Physics, Bhavan’s Sheth R. A. College of Science, Ahmedabad, Gujarat - 380001, India; c Research Centre for Crystalline Materials, School of Science and Technology, Sunway University, 47500 Bandar Sunway, Selangor Darul Ehsan, Malaysia

**Keywords:** crystal structure, Schiff base, Hirshfeld surface analysis, computational chemistry

## Abstract

The title Schiff base compound, C_14_H_10_Cl_2_N_2_O, features an *E* configuration about each of the C=N imine bonds. Overall, the mol­ecule is approximately planar with the dihedral angle between the central C_2_N_2_ residue (r.m.s. deviation = 0.0371 Å) and the peripheral hy­droxy­benzene and chloro­benzene rings being 4.9 (3) and 7.5 (3)°, respectively. Nevertheless, a small twist is evident about the central N—N bond [the C—N—N—C torsion angle = −172.7 (2)°]. An intra­molecular hy­droxy-O—H⋯N(imine) hydrogen bond closes an *S*(6) loop. In the crystal, π–π stacking inter­actions between hy­droxy- and chloro­benzene rings [inter-centroid separation = 3.6939 (13) Å] lead to a helical supra­molecular chain propagating along the *b*-axis direction; the chains pack without directional inter­actions between them. The calculated Hirshfeld surfaces point to the importance of H⋯H and Cl⋯H/H⋯Cl contacts to the overall surface, each contributing approximately 29% of all contacts. However, of these only Cl⋯H contacts occur at separations less than the sum of the van der Waals radii. The aforementioned π–π stacking inter­actions contribute 12.0% to the overall surface contacts. The calculation of the inter­action energies in the crystal indicates significant contributions from the dispersion term.

## Abstract 2

An *E* configuration about each of the two imine bonds is found in the title mol­ecule which, to a first approximation, is planar. The main feature of the mol­ecular packing is π–π stacking leading to helical, supra­molecular chains.

## Chemical context   

Being deprotonable and readily substituted with various residues, Schiff base mol­ecules are prominent as multidentate ligands for the generation of a wide variety of metal complexes. In our laboratory, a key motivation for studies in this area arises from our interest in the Schiff bases themselves and of their metal complexes, which are well-known to possess a wide spectrum of biological activity against disease-causing microorganisms (Tian *et al.*, 2009 ▸; 2011 ▸). Over and beyond biological considerations, Schiff bases are also suitable for the development of non-linear optical materials because of their solvato-chromaticity (Labidi, 2013 ▸).
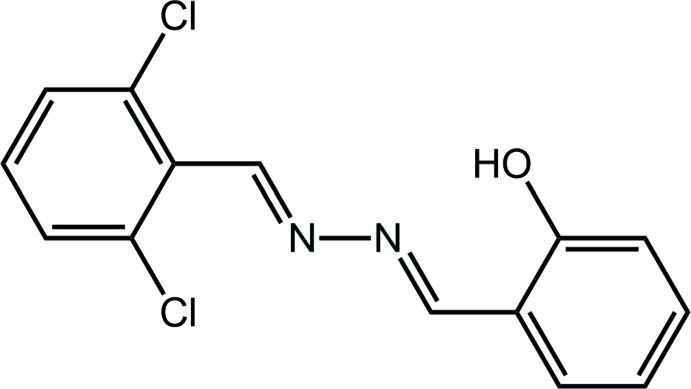


As reported recently, the title compound, (I), a potentially multidentate ligand has anti-bacterial and anti-fungal action against a range of microorganisms (Manawar *et al.*, 2019 ▸). As a part of complementary structural studies on these mol­ecules, the crystal and mol­ecular structures of (I) are described herein together with a detailed analysis of the calculated Hirshfeld surfaces.

## Structural commentary   

**Figure 1:**
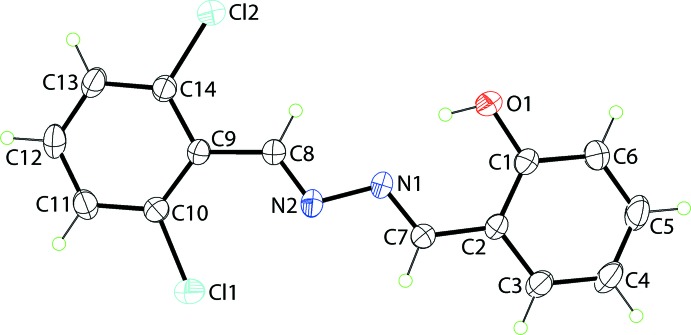
The mol­ecular structure of (I) showing the atom-labelling scheme and displacement ellipsoids at the 35% probability level.

The title Schiff base mol­ecule (I), Fig. 1 ▸, features two imine bonds, C7=N1 [1.281 (2) Å] and C8=N2 [1.258 (3) Å] with the configuration about each being *E*. The central N1, N2, C7, C8 chromophore is close to being the planar, exhibiting an r.m.s. deviation of 0.0371 Å, with deviations of 0.0390 (11) and 0.0372 (10) Å above and below the means plane for the N1 and C7 atoms, respectively. There is a small but significant twist about the central N1—N2 bond [1.405 (2) Å] as seen in the value of the C7—N1—N2—C8 torsion angle of −172.7 (2)°. The dihedral angles between the central plane and those through the hy­droxy­benzene [4.9 (3)°] and chloro­benzene [7.5 (3)°] rings, respectively, and that between the outer rings [4.83 (13)°] indicate that to a first approximation, the entire mol­ecule is planar. An intra­molecular hy­droxy-O—H⋯N(imine) hydrogen bond is noted, Table 1 ▸, which closes an *S*(6) loop.

## Supra­molecular features   

**Figure 2:**
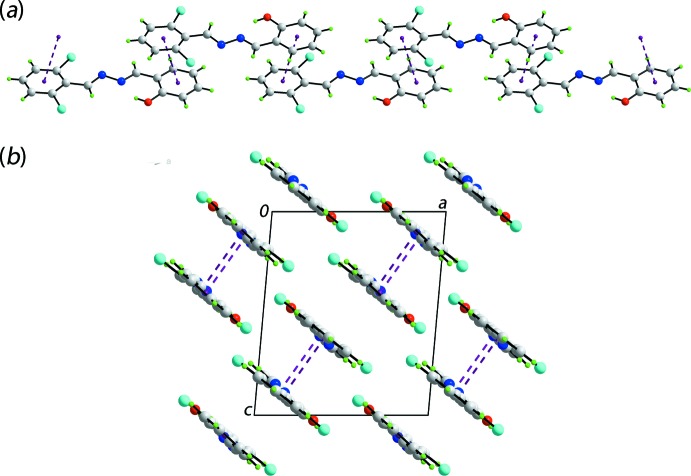
Mol­ecular packing in the crystal of (I): (*a*) supra­molecular chain sustained by π(hy­droxy­benzene)–π(chloro­benzene) inter­actions shown as purple dashed lines and (*b*) a view of the unit-cell contents in a projection down the *b* axis.

The most prominent supra­molecular association in the crystal of (I) are π–π stacking inter­actions. These occur between the hy­droxy- and chloro­benzene rings with an inter-centroid separation = 3.6939 (13) Å and angle of inclination = 4.32 (11)° [symmetry operation 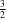 − *x*, 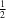 + *y*, 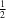 − *z*]. As these inter­actions occur at both ends of the mol­ecule and are propagated by screw-symmetry (2_1_), the topology of the resultant chain is helical, Fig. 2 ▸(*a*). According to the criteria incorporated in *PLATON* (Spek, 2009 ▸), there are no directional inter­actions connecting chains; a view of the unit-cell contents is shown in Fig. 2 ▸(*b*). The presence of other, weaker points of contact between atoms and between residues are noted – these are discussed in more detail in *Hirshfeld surface analysis.*

## Hirshfeld surface analysis   

**Figure 3:**
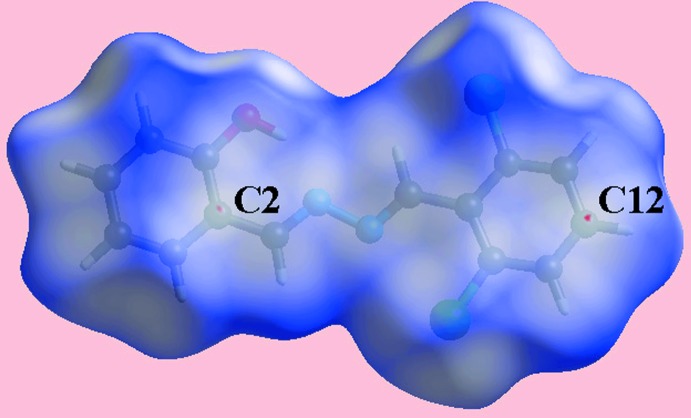
A view of the Hirshfeld surface for (I) mapped over *d*_norm_ in the range −0.001 to + 1.301 (arbitrary units), highlighting diminutive red spots near the C2 and C12 atoms owing to their participation in C⋯C contacts.

**Figure 4:**
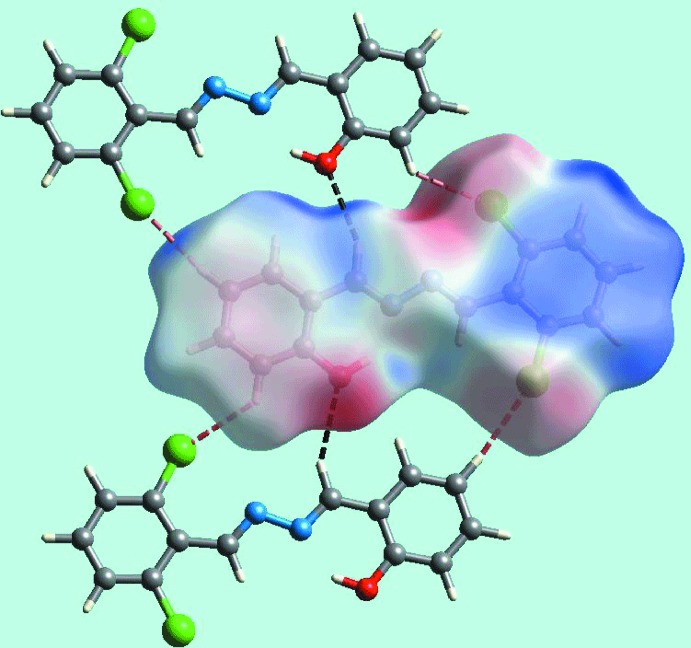
A view of the Hirshfeld surface mapped over the electrostatic potential (the red and blue regions represent negative and positive electrostatic potentials, respectively) in the range −0.065 to + 0.039 atomic units, with short inter­atomic Cl⋯H and O⋯H contacts highlighted with red and black dashed dashed lines, respectively.

**Figure 5:**
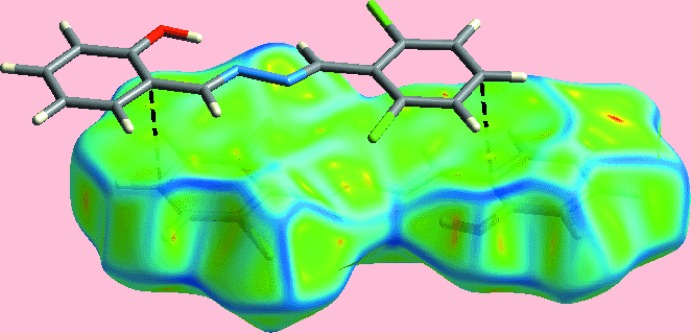
A view of Hirshfeld surface mapped with curvedness showing edge-to-edge π–π overlap through black dashed lines.

The Hirshfeld surface calculations for (I) were performed employing *Crystal Explorer 17* (Turner *et al.*, 2017 ▸) and recently published protocols (Tan *et al.*, 2019 ▸). On the Hirshfeld surface mapped over *d*_norm_ in Fig. 3 ▸, the short inter­atomic contact between the hy­droxy­phenyl-C2 and chloro­phenyl-C12 atoms (Table 2 ▸) is characterized as small red spots near them. The Cl1 and Cl2 atoms form short intra-layer Cl⋯H contacts with the H4 and H6 atoms of the hy­droxy­phenyl ring (Table 2 ▸) and are represented in Fig. 4 ▸, showing a reference mol­ecule within the Hirshfeld surface mapped over the electrostatic potential. The Hirshfeld surface mapped with curvedness is shown in Fig. 5 ▸, which highlights the influence of the short inter­atomic C⋯C contacts in the packing (Table 2 ▸) consistent with the edge-to-edge π–π stacking between symmetry related mol­ecules.

**Figure 6:**
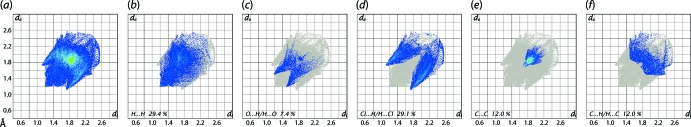
(*a*) A comparison of the full two-dimensional fingerprint plot for (I) and those delineated into (*b*) H⋯H, (*c*) O⋯H/H⋯O, (*d*) Cl⋯H/H⋯Cl, (*e*) C⋯C and (*f*) C⋯H/H⋯C contacts.

The full two-dimensional fingerprint plot for (I), Fig. 6 ▸(*a*), and those decomposed into H⋯H, O⋯H/H⋯O, Cl⋯H/H⋯Cl, C⋯C and C⋯H/H⋯C contacts are illustrated in Fig. 6 ▸(*b*)-(*f*), respectively. The percentage contributions from the different inter­atomic contacts to the Hirshfeld surface of (I) are qu­anti­tatively summarized in Table 3 ▸. It is evident from the fingerprint plot delineated into H⋯H contacts in Fig. 6 ▸(*b*) that their inter­atomic distances are equal to or greater than the sum of their respective van der Waals radii. The fingerprint plot delineated into O⋯H/H⋯O contacts in Fig. 6 ▸(*c*) indicates the presence of short inter­atomic O⋯H contacts involving hy­droxy-O1 and phenyl-H7 atoms through the pair of forceps-like tips at *d*_e_ + *d*_i_ < 2.7 Å. The presence of a pair of conical tips at *d*_e_ + *d*_i_ ∼2.9 Å in the fingerprint plot delineated into Cl⋯H/H⋯Cl contacts in Fig. 6 ▸(*d*) are due to the Cl⋯H contacts listed in Table 2 ▸. In the fingerprint plot decomposed into C⋯C contacts in Fig. 6 ▸(*e*), the π–π stacking between symmetry-related hy­droxy- and chloro­benzene rings are characterized as the pair of small forceps-like tips at *d*_e_ + *d*_i_ ∼3.4 Å together with the green points distributed around *d*_e_ = *d*_i_ ∼1.8 Å. The fingerprint plot delineated into C⋯H/H⋯ C contacts in Fig. 6 ▸(*f*) confirms the absence of significant C—H⋯ π and C⋯H/H⋯C contacts as the points in the respective delineated plot are distributed farther than sum of their respective van der Waals radii. The small contribution from other inter­atomic contacts to the Hirshfeld surfaces of (I) summarized in Table 3 ▸ have a negligible effect on the mol­ecular packing.

## Computational chemistry   

**Figure 7:**
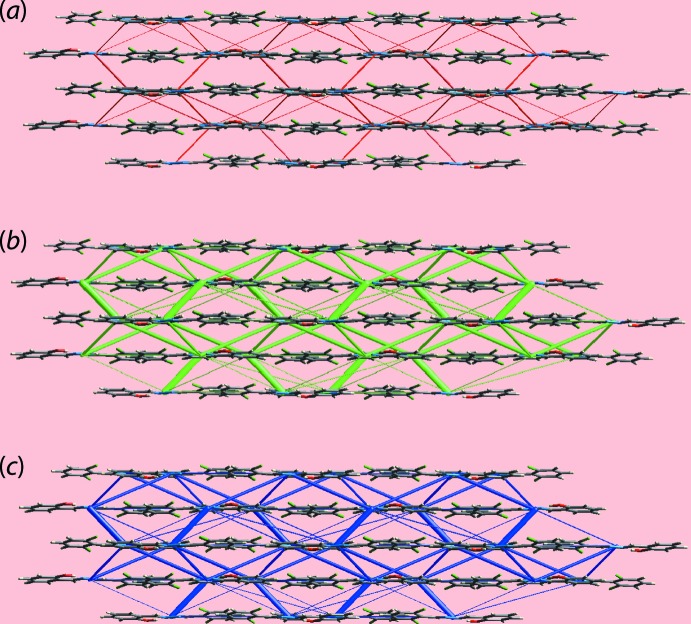
The energy frameworks calculated for (I) showing the (*a*) electrostatic potential force, (*b*) dispersion force and (*c*) total energy. The energy frameworks were adjusted to the same scale factor of 50 with a cut-off value of 5 kJ mol^−1^ within 4 × 4 × 4 unit cells

In the present analysis, the pairwise inter­action energies between the mol­ecules in the crystal were calculated by summing up four different energy components (Turner *et al.*, 2017 ▸). These comprise electrostatic (*E*_ele_), polarization (*E*_pol_), dispersion (*E*_dis_) and exchange–repulsion (*E*_rep_), and were obtained using the wave function calculated at the B3LYP/6-31G(d,p) level of theory. From the inter­molecular inter­action energies collated in Table 4 ▸, it is apparent that the dispersion energy component has a major influence in the formation of the supra­molecular architecture of (I) as conventional hydrogen bonding is absent. The energy associated with the π–π stacking inter­action between symmetry-related hy­droxy- and chloro­benzene rings is greater than the energy calculated for the Cl⋯H/H⋯Cl and O⋯H/H⋯O contacts. The magnitudes of inter­molecular energies were also represented graphically in Fig. 7 ▸ by energy frameworks whereby the cylinders join the centroids of mol­ecular pairs using a red, green and blue colour scheme for the *E*_ele_, *E*_disp_ and *E*_tot_ components, respectively; the radius of the cylinder is proportional to the magnitude of inter­action energy.

## Database survey   

Given the great inter­est in Schiff bases and their complexation to transition metals and other heavy elements, it is not surprising that there is a wealth of structural data for these compounds in the Cambridge Structural Database (CSD; Groom *et al.*, 2016 ▸). Indeed, there are over 150 ‘hits’ for the basic framework 2-OH-C_6_—C=N—N=C—C_6_ featured in (I). This number is significantly reduced when H atoms are added to the imine-carbon atoms and examples where a second hy­droxy substituent present in the 2-position of the phenyl ring is excluded. Thus, there are eight mol­ecules in the CSD containing the fragment 2-OH-C_6_-C(H)=N—N=C(H)-C_6_, excluding two calix(4)arene derivatives. While the formation of the hy­droxy-O—H⋯N(imine) bond is common to all mol­ecules, there is a certain degree of conformational flexibility in the mol­ecules as seen in the relevant geometric data collated in Table 5 ▸. From the data in Table 5 ▸, the mol­ecule reported herein, *i.e*. (I), exhibits the greatest twist about the central N—N bond, whereas virtually no twist is seen in the central C—N—N—C torsion angle for (V), *i.e*. −179.8 (2)°. The dihedral angles between the central C_2_N_2_ residue and the hy­droxy-substituted benzene ring span a range 2.27 (9)°, again in (V), to 10.58 (4)°, for (IV). A significantly greater range is noted in the dihedral angles between C_2_N_2_ and the second benzene ring, *i.e*. 2.32 (12)° in (VII) to 29.03 (16)° in (II). Accordingly, the greatest deviation from co-planarity among the nine mol­ecules included in Table 5 ▸ is found in (II) where the dihedral angle between the outer rings is 31.35 (8)°.

## Synthesis and crystallization   

Compound (I) was prepared as reported in the literature from the condensation reaction of 2,6-di­chloro­benzaldehyde and hydrazine hydrate (Manawar *et al.*, 2019 ▸). Crystals in the form of light-yellow blocks for the X-ray study were grown by the slow evaporation of its chloro­form solution.

## Refinement   

Crystal data, data collection and structure refinement details are summarized in Table 6 ▸. Carbon-bound H-atoms were placed in calculated positions (C—H = 0.93 Å) and were included in the refinement in the riding-model approximation, with *U*_iso_(H) set to 1.2*U*_eq_(C). The position of the O-bound H atom was refined with *U*_iso_(H) set to 1.5*U*_eq_(O).

## Supplementary Material

Crystal structure: contains datablock(s) I, global. DOI: 10.1107/S2056989019012349/hb7852sup1.cif

Structure factors: contains datablock(s) I. DOI: 10.1107/S2056989019012349/hb7852Isup2.hkl

CCDC reference: 1857868

Additional supporting information:  crystallographic information; 3D view; checkCIF report

## Figures and Tables

**Table 1:** Hydrogen-bond geometry (Å, °)

*D*—H⋯*A*	*D*—H	H⋯*A*	*D*⋯*A*	*D*—H⋯*A*
O1—H1O⋯N1	0.87 (3)	1.87 (3)	2.632 (2)	147 (3)

**Table 2:** Summary of short inter­atomic contacts (Å) in (I)^*a*^

Contact	Distance	Symmetry operation
Cl1⋯H6	2.86	− 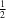 + *x*, 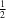 − *y*, − 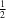 + *z*
Cl2⋯H4	2.85	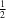 + *x*, 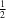 − *y*, 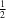 + *z*
O1⋯H7	2.68	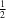 + *x*, 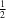 − *y*, 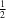 + *z*
C2⋯C12	3.399 (3)	1 − *x*, − *y*, 1 − *z*

Note: (*a*) The inter­atomic distances were calculated using *Crystal Explorer 17* (Turner *et al.*, 2017 ▸) whereby the *X*—H bond lengths are adjusted to their neutron values.

**Table 3:** Percentage contributions of inter­atomic contacts to the Hirshfeld surface for (I)

Contact	Percentage contribution
H⋯H	29.4
Cl⋯H/H⋯Cl	29.1
O⋯H/H⋯O	7.4
C⋯H/H⋯C	12.0
C⋯C	12.0
N⋯H/H⋯N	4.5
C⋯N/N⋯C	3.9
C⋯Cl/Cl⋯C	0.6
Cl⋯Cl	0.4
Cl⋯N/N⋯Cl	0.4
Cl⋯O/O⋯Cl	0.1
C⋯O/O⋯C	0.1

**Table 4:** Summary of inter­action energies (kJ mol^−1^) calculated for (I)

Contact	*R* (Å)	*E*_ele_	*E*_pol_	*E*_dis_	*E*_rep_	*E*_tot_
C2⋯C12^i^	4.00	−13.1	−1.4	−77.2	42.7	−55.8
*Cg*(C1–C6)⋯*Cg*(C9–C14)^ii^	8.58	−5.9	−0.9	−40.1	20.6	−29.2
Cl1⋯H6^iii^ +						
Cl2⋯H4^iv^ +	8.53	−10.4	−1.8	−20.9	19.1	−18.7
O1⋯H7^iv^						

Symmetry codes: (i) 1 − *x*, −*y*, 1 − *z*; (ii) 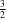 − *x*, 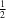 + *y*, 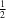 − *z*; (iii) −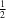 + *x*, 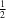 − *y*, −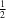 + *z*; (iv) 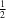 + *x*, 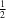 − *y*, 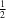 + *z*.

**Table 5:** Geometric data (°) for related 2-OH—C_6_—C(H)=N—N=C(H)—C_6_ mol­ecules, *i.e. R* ^1^—C(H)=N—N=C(H)—*R* ^2^

Compound	*R* ^1^	*R* ^2^	C—N—N—C	C_2_N_2_/*R*-C_6_	C_2_N_2_/*R*′-C_6_	*R*-C_6_/*R*′-C_6_	REFCODE
(I)	2-OH—C_6_H_4_	2,6-Cl_2_—C_6_H_3_	−172.7 (2)	4.9 (3)	7.5 (3)	4.83 (13)	–*^*a*^*
(II)	2-OH—C_6_H_4_	anthracen-9-yl	179.1 (2)	2.84 (13)	29.03 (16)	31.35 (8)	KOBXAD*^*b*^*
(III)	2-OH—C_6_H_4_	2-EtOC(=O)CH_2_—C_6_H_4_	173.32 (14)	7.25 (9)	20.02 (9)	27.26 (5)	LOSJIO*^*c*^*
(IV)	2,3-(OH)_2_-4,6-(*t*-Bu)_2_—C_6_H	4-Me_2_NC_6_H_4_	−178.09 (12)	10.58 (4)	4.61 (4)	15.03 (3)	EDIQOA*^*d*^*
(V)	2-naphthol	4-Me_2_N—C_6_H_4_	−179.8 (2)	2.27 (9)	6.49 (13)	7.84 (6)	EZUYEF*^*e*^*
(VI)	2-naphthol	4-OH—C_6_H_4_	179.30 (16)	3.93 (12)	8.44 (12)	11.91 (6)	RUTGEU*^*f*^*
(VII)	2-naphthol	4-Me_2_N—C_6_H_4_	177.98 (15)	4.90 (10)	2.32 (12)	3.82 (6)	RUTFET*^*g*^*
(VIII*	2-naphthol	4-OH-3-MeO-C_6_H_4_	178.73 (14)	5.78 (10)	15.06 (7)	13.14 (5)	POMNIQ*^*h*^*
			177.74 (15)	6.65 (9)	12.05 (11)	18.46 (6)	
(IX)*	2-naphthol	pyren-1-yl	−173 (1)	2.6 (8)	4.4 (7)	6.9 (4)	APACEB^*i*^
			173 (1)	5.3 (7)	4.7 (7)	7.9 (4)	

* Two independent mol­ecules in the asymmetric unit. References: (*a*) This work; (*b*) Patil & Das (2017 ▸); (*c*) Akkurt *et al.* (2015 ▸); (*d*) Arsenyev *et al.* (2016 ▸); (*e*) Ghosh, Adhikari *et al.* (2016 ▸); (*f*) Ghosh, Ta *et al.* (2016 ▸); (*g*) Ghosh, Ta *et al.* (2016 ▸); (*h*) Kumari *et al.* (2014 ▸); (*i*) Ghosh, Ganguly *et al.* (2016 ▸)

**Table 6:** Experimental details

Crystal data	
Chemical formula	C_14_H_10_Cl_2_N_2_O
*M*_r_	293.14
Crystal system, space group	Monoclinic, *P*2_1_/*n*
Temperature (K)	296
*a*, *b*, *c* (Å)	8.5614 (8), 15.6055 (12), 10.0527 (9)
β (°)	95.031 (3)
*V* (Å^3^)	1337.9 (2)
*Z*	4
Radiation type	Mo *K*α
μ (mm^−1^)	0.48
Crystal size (mm)	0.35 × 0.30 × 0.30

Data collection	
Diffractometer	Bruker Kappa APEXII CCD
Absorption correction	Multi-scan (*SADABS*; Bruker, 2004 ▸)
*T*_min_, *T*_max_	0.846, 0.867
No. of measured, independent and observed [*I* > 2σ(*I*)] reflections	10171, 3185, 2244
*R*_int_	0.023
(sin θ/λ)_max_ (Å^−1^)	0.666

Refinement	
*R*[*F* ^2^ > 2σ(*F* ^2^)], *wR*(*F* ^2^), *S*	0.044, 0.138, 1.05
No. of reflections	3185
No. of parameters	175
H-atom treatment	H atoms treated by a mixture of independent and constrained refinement
Δρ_max_, Δρ_min_ (e Å^−3^)	0.37, −0.28

Computer programs: *APEX2* and *SAINT* (Bruker, 2004 ▸), *SIR92* (Altomare *et al.*, 1994 ▸), *SHELXL2014* (Sheldrick, 2015 ▸), *ORTEP-3 for Windows* (Farrugia, 2012 ▸), *DIAMOND* (Brandenburg, 2006 ▸) and *publCIF* (Westrip, 2010 ▸).

## supplementary crystallographic information

### Crystal data

**Table:**

C_14_H_10_Cl_2_N_2_O	*F*(000) = 600
*M**_r_* = 293.14	*D*_x_ = 1.455 Mg m^−^^3^
Monoclinic, *P*2_1_/*n*	Mo *K*α radiation, λ = 0.71073 Å
*a* = 8.5614 (8) Å	Cell parameters from 4447 reflections
*b* = 15.6055 (12) Å	θ = 4.8–56.3°
*c* = 10.0527 (9) Å	µ = 0.48 mm^−^^1^
β = 95.031 (3)°	*T* = 296 K
*V* = 1337.9 (2) Å^3^	Block, light-yellow
*Z* = 4	0.35 × 0.30 × 0.30 mm

### Data collection

**Table:**

Bruker Kappa APEXII CCD diffractometer	2244 reflections with *I* > 2σ(*I*)
Radiation source: X-ray tube	*R*_int_ = 0.023
ω and φ scan	θ_max_ = 28.3°, θ_min_ = 2.6°
Absorption correction: multi-scan (SADABS; Bruker, 2004)	*h* = −11→11
*T*_min_ = 0.846, *T*_max_ = 0.867	*k* = −15→20
10171 measured reflections	*l* = −11→12
3185 independent reflections	

### Refinement

**Table:**

Refinement on *F*^2^	Primary atom site location: structure-invariant direct methods
Least-squares matrix: full	Secondary atom site location: difference Fourier map
*R*[*F*^2^ > 2σ(*F*^2^)] = 0.044	Hydrogen site location: mixed
*wR*(*F*^2^) = 0.138	H atoms treated by a mixture of independent and constrained refinement
*S* = 1.05	*w* = 1/[σ^2^(*F*_o_^2^) + (0.069*P*)^2^ + 0.4482*P*] where *P* = (*F*_o_^2^ + 2*F*_c_^2^)/3
3185 reflections	(Δ/σ)_max_ < 0.001
175 parameters	Δρ_max_ = 0.37 e Å^−^^3^
0 restraints	Δρ_min_ = −0.28 e Å^−^^3^

### Special details

**Table:**

Geometry. All esds (except the esd in the dihedral angle between two l.s. planes) are estimated using the full covariance matrix. The cell esds are taken into account individually in the estimation of esds in distances, angles and torsion angles; correlations between esds in cell parameters are only used when they are defined by crystal symmetry. An approximate (isotropic) treatment of cell esds is used for estimating esds involving l.s. planes.

### Fractional atomic coordinates and isotropic or equivalent isotropic displacement parameters (Å^2^)

**Table:**

	*x*	*y*	*z*	*U*_iso_*/*U*_eq_	
Cl1	0.37913 (8)	−0.06610 (4)	0.23254 (7)	0.0669 (2)	
Cl2	0.90894 (8)	−0.10632 (4)	0.57108 (7)	0.0646 (2)	
O1	0.8423 (2)	0.25607 (11)	0.52671 (19)	0.0636 (5)	
H3O	0.808 (4)	0.206 (2)	0.501 (3)	0.095*	
N1	0.66249 (19)	0.14415 (10)	0.39038 (17)	0.0395 (4)	
N2	0.6061 (2)	0.06313 (10)	0.34827 (19)	0.0462 (5)	
C1	0.7508 (2)	0.31651 (12)	0.4623 (2)	0.0406 (5)	
C2	0.6261 (2)	0.29483 (12)	0.36883 (19)	0.0343 (4)	
C3	0.5366 (3)	0.36160 (13)	0.3082 (2)	0.0449 (5)	
H3	0.4534	0.3486	0.2457	0.054*	
C4	0.5688 (3)	0.44563 (15)	0.3390 (2)	0.0555 (6)	
H4	0.5073	0.4890	0.2984	0.067*	
C5	0.6932 (3)	0.46555 (14)	0.4305 (3)	0.0590 (7)	
H5	0.7160	0.5226	0.4505	0.071*	
C6	0.7839 (3)	0.40175 (14)	0.4924 (3)	0.0535 (6)	
H6	0.8671	0.4158	0.5543	0.064*	
C7	0.5859 (2)	0.20707 (12)	0.3350 (2)	0.0378 (4)	
H7	0.5026	0.1960	0.2716	0.045*	
C8	0.6741 (2)	0.00413 (12)	0.4161 (2)	0.0397 (5)	
H8	0.7496	0.0206	0.4834	0.048*	
C9	0.6461 (2)	−0.08822 (11)	0.39956 (18)	0.0333 (4)	
C10	0.5215 (2)	−0.12658 (13)	0.3210 (2)	0.0389 (5)	
C11	0.5039 (3)	−0.21481 (14)	0.3119 (2)	0.0453 (5)	
H11	0.4196	−0.2381	0.2594	0.054*	
C12	0.6111 (3)	−0.26778 (13)	0.3805 (2)	0.0487 (5)	
H12	0.5998	−0.3269	0.3734	0.058*	
C13	0.7354 (3)	−0.23366 (13)	0.4599 (2)	0.0459 (5)	
H13	0.8080	−0.2694	0.5064	0.055*	
C14	0.7504 (2)	−0.14562 (12)	0.4692 (2)	0.0387 (5)	

### Atomic displacement parameters (Å^2^)

**Table:**

	*U*^11^	*U*^22^	*U*^33^	*U*^12^	*U*^13^	*U*^23^
Cl1	0.0580 (4)	0.0514 (4)	0.0850 (5)	−0.0036 (3)	−0.0299 (3)	0.0108 (3)
Cl2	0.0631 (4)	0.0443 (3)	0.0800 (5)	0.0068 (3)	−0.0304 (3)	−0.0017 (3)
O1	0.0630 (11)	0.0375 (8)	0.0835 (13)	0.0107 (8)	−0.0322 (9)	−0.0042 (8)
N1	0.0445 (9)	0.0262 (8)	0.0470 (10)	0.0012 (7)	−0.0009 (8)	0.0012 (7)
N2	0.0586 (11)	0.0274 (8)	0.0503 (11)	−0.0011 (8)	−0.0076 (9)	−0.0015 (7)
C1	0.0429 (11)	0.0321 (10)	0.0461 (12)	0.0060 (8)	0.0000 (9)	−0.0012 (8)
C2	0.0393 (10)	0.0284 (9)	0.0356 (10)	0.0048 (8)	0.0047 (8)	0.0015 (8)
C3	0.0543 (12)	0.0381 (11)	0.0414 (11)	0.0098 (9)	−0.0003 (10)	0.0031 (9)
C4	0.0782 (17)	0.0357 (11)	0.0520 (14)	0.0177 (11)	0.0023 (12)	0.0066 (10)
C5	0.0847 (18)	0.0287 (10)	0.0629 (15)	0.0041 (11)	0.0026 (13)	−0.0043 (10)
C6	0.0620 (15)	0.0364 (11)	0.0598 (14)	−0.0004 (10)	−0.0085 (12)	−0.0088 (10)
C7	0.0413 (10)	0.0338 (10)	0.0373 (10)	0.0012 (8)	−0.0020 (8)	0.0001 (8)
C8	0.0389 (10)	0.0315 (9)	0.0478 (12)	−0.0012 (8)	−0.0020 (9)	0.0005 (9)
C9	0.0376 (10)	0.0292 (9)	0.0333 (10)	0.0001 (7)	0.0051 (8)	0.0013 (7)
C10	0.0419 (11)	0.0359 (10)	0.0381 (10)	−0.0009 (8)	−0.0005 (8)	0.0033 (8)
C11	0.0551 (13)	0.0380 (11)	0.0424 (12)	−0.0082 (9)	0.0017 (10)	−0.0042 (9)
C12	0.0688 (15)	0.0285 (10)	0.0496 (13)	−0.0054 (10)	0.0106 (11)	−0.0019 (9)
C13	0.0608 (14)	0.0309 (10)	0.0459 (12)	0.0078 (9)	0.0041 (10)	0.0041 (9)
C14	0.0444 (11)	0.0331 (10)	0.0382 (11)	0.0016 (8)	0.0021 (9)	0.0006 (8)

### Geometric parameters (Å, º)

**Table:**

Cl1—C10	1.725 (2)	C5—C6	1.377 (3)
Cl2—C14	1.739 (2)	C5—H5	0.9300
O1—C1	1.354 (2)	C6—H6	0.9300
O1—H3O	0.87 (3)	C7—H7	0.9300
N1—C7	1.281 (2)	C8—C9	1.468 (3)
N1—N2	1.405 (2)	C8—H8	0.9300
N2—C8	1.258 (3)	C9—C14	1.407 (3)
C1—C6	1.388 (3)	C9—C10	1.405 (3)
C1—C2	1.401 (3)	C10—C11	1.387 (3)
C2—C3	1.401 (3)	C11—C12	1.375 (3)
C2—C7	1.445 (3)	C11—H11	0.9300
C3—C4	1.370 (3)	C12—C13	1.379 (3)
C3—H3	0.9300	C12—H12	0.9300
C4—C5	1.380 (4)	C13—C14	1.382 (3)
C4—H4	0.9300	C13—H13	0.9300
			
C1—O1—H3O	109 (2)	C2—C7—H7	119.3
C7—N1—N2	114.17 (16)	N2—C8—C9	126.41 (18)
C8—N2—N1	111.38 (17)	N2—C8—H8	116.8
O1—C1—C6	117.72 (19)	C9—C8—H8	116.8
O1—C1—C2	121.84 (17)	C14—C9—C10	115.23 (17)
C6—C1—C2	120.44 (19)	C14—C9—C8	118.56 (17)
C3—C2—C1	117.91 (18)	C10—C9—C8	126.20 (17)
C3—C2—C7	119.52 (18)	C11—C10—C9	122.19 (18)
C1—C2—C7	122.57 (17)	C11—C10—Cl1	116.18 (16)
C4—C3—C2	121.5 (2)	C9—C10—Cl1	121.61 (15)
C4—C3—H3	119.3	C12—C11—C10	120.0 (2)
C2—C3—H3	119.3	C12—C11—H11	120.0
C3—C4—C5	119.6 (2)	C10—C11—H11	120.0
C3—C4—H4	120.2	C13—C12—C11	120.35 (18)
C5—C4—H4	120.2	C13—C12—H12	119.8
C6—C5—C4	120.7 (2)	C11—C12—H12	119.8
C6—C5—H5	119.7	C12—C13—C14	119.0 (2)
C4—C5—H5	119.7	C12—C13—H13	120.5
C5—C6—C1	119.9 (2)	C14—C13—H13	120.5
C5—C6—H6	120.1	C13—C14—C9	123.19 (19)
C1—C6—H6	120.1	C13—C14—Cl2	117.00 (16)
N1—C7—C2	121.47 (18)	C9—C14—Cl2	119.80 (14)
N1—C7—H7	119.3		
			
C7—N1—N2—C8	−172.7 (2)	N2—C8—C9—C14	169.6 (2)
O1—C1—C2—C3	179.3 (2)	N2—C8—C9—C10	−11.4 (4)
C6—C1—C2—C3	−0.3 (3)	C14—C9—C10—C11	−0.6 (3)
O1—C1—C2—C7	0.3 (3)	C8—C9—C10—C11	−179.6 (2)
C6—C1—C2—C7	−179.4 (2)	C14—C9—C10—Cl1	178.08 (15)
C1—C2—C3—C4	−0.2 (3)	C8—C9—C10—Cl1	−0.9 (3)
C7—C2—C3—C4	178.9 (2)	C9—C10—C11—C12	−0.4 (3)
C2—C3—C4—C5	0.8 (4)	Cl1—C10—C11—C12	−179.23 (18)
C3—C4—C5—C6	−0.9 (4)	C10—C11—C12—C13	0.8 (3)
C4—C5—C6—C1	0.3 (4)	C11—C12—C13—C14	0.0 (3)
O1—C1—C6—C5	−179.4 (2)	C12—C13—C14—C9	−1.1 (3)
C2—C1—C6—C5	0.3 (4)	C12—C13—C14—Cl2	179.81 (17)
N2—N1—C7—C2	178.69 (18)	C10—C9—C14—C13	1.4 (3)
C3—C2—C7—N1	−178.4 (2)	C8—C9—C14—C13	−179.5 (2)
C1—C2—C7—N1	0.6 (3)	C10—C9—C14—Cl2	−179.53 (15)
N1—N2—C8—C9	−179.54 (19)	C8—C9—C14—Cl2	−0.5 (3)

### Hydrogen-bond geometry (Å, º)

**Table:**

*D*—H···*A*	*D*—H	H···*A*	*D*···*A*	*D*—H···*A*
O1—H1O···N1	0.87 (3)	1.87 (3)	2.632 (2)	147 (3)
